# Spontaneous pulmonary hematoma with diffuse pulmonary ossification requiring emergency treatment: a case report

**DOI:** 10.1186/s40792-019-0589-6

**Published:** 2019-02-18

**Authors:** Takumi Sonokawa, Takuma Matsui, Kyoshiro Takegahara, Tatsuya Inoue, Takeru Kashiwada, Yasuhiro Terasaki, Jitsuo Usuda

**Affiliations:** 1 Department of Thoracic Surgery, Nippon Medical School Hospital, 1-1-5 Sendagi, Bunkyo-ku, Tokyo, 113-8603 Japan; 2 Department of Pulmonary Medicine and Oncology, Graduate School of Medicine, Nippon Medical School, Tokyo, 113-8603 Japan; 3 Department of Analytic Human Pathology, Nippon Medical School, 1-1-5 Sendagi, Bunkyo-ku, Tokyo, 113-8603 Japan

**Keywords:** Pulmonary hematoma, Lung tumor, Diffuse pulmonary ossification, Intrathoracic hemorrhage

## Abstract

**Background:**

Pulmonary hematoma is most often caused by thoracic trauma. However, rare cases of pulmonary hematoma without any obvious cause are sometimes reported, when the condition is referred to as spontaneous or idiopathic pulmonary hematoma. Herein, we report a very rare case—to the best of our knowledge, this is the first case of pulmonary hematoma associated with diffuse pulmonary ossification requiring emergency treatment reported in the English literature.

**Case presentation:**

Our patient was a 44-year-old man with a history of IgA nephropathy and had no history of trauma or anticoagulant use. He presented with the chief complaint of bloody sputum and was referred to our hospital for detailed examination. Chest computed tomography showed a right pleural effusion and a large round mass in the right lower lobe. We performed chest tube drainage of the right thorax and confirmed bloody pleural effusion. Although bronchial artery embolization was performed, the patient’s anemia worsened, and we performed right lower lobectomy. Histopathological examination of the resected specimen showed a hematoma with diffuse pulmonary ossification, although the relationship between the two was unclear. There was no evidence of malignancy or angiitis. Therefore, we made the diagnosis of spontaneous pulmonary hematoma in this case. The postoperative course was uneventful. The patient is currently under observation as an outpatient, and a recent chest X-ray showed no evidence of recurrence.

**Conclusion:**

We report a case of spontaneous pulmonary hematoma with diffuse pulmonary ossification. Although the relationship between the two remains unclear, considering the possibility of appearance of new lesions/recurrence, we believe that careful follow-up is necessary for this patient.

## Background

Pulmonary hematoma refers to the presence of blood within the alveolar and interstitial spaces [1]. In general, intrathoracic and/or pulmonary hematomas are caused by thoracic trauma. However, rare cases without obvious cause have been reported, when the condition is referred to as spontaneous or idiopathic pulmonary hematoma. Because pulmonary hematomas often resolve spontaneously over time, they do not require any aggressive treatment. We report this interesting case of spontaneous pulmonary hematoma with diffuse pulmonary ossification in which emergency treatment was required.

## Case presentation

**Fig. 1:**
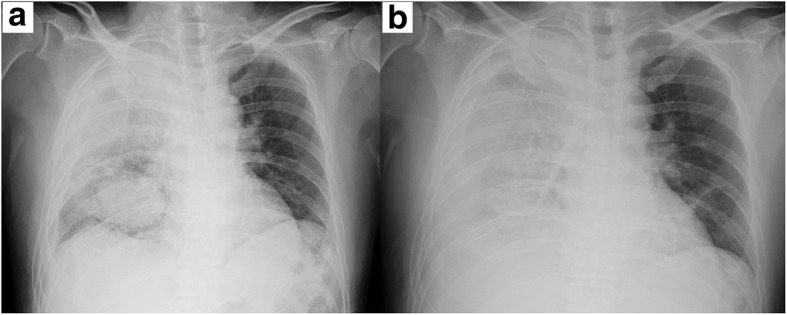
Chest X-ray showing reduced radiolucency of the right lung field and an opacity in the right lower lung field (**a**). The following day, the chest X-ray findings deteriorated further (**b**)

**Fig. 2:**
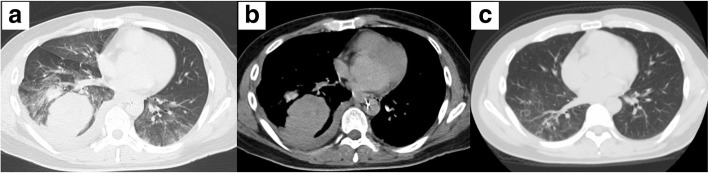
Chest computed tomographic images revealing right pleural effusion and a large round mass in the right lower lobe (**a**, **b**). This mass was not observed in a CT examination performed 10 months earlier (**c**)

**Fig. 3:**
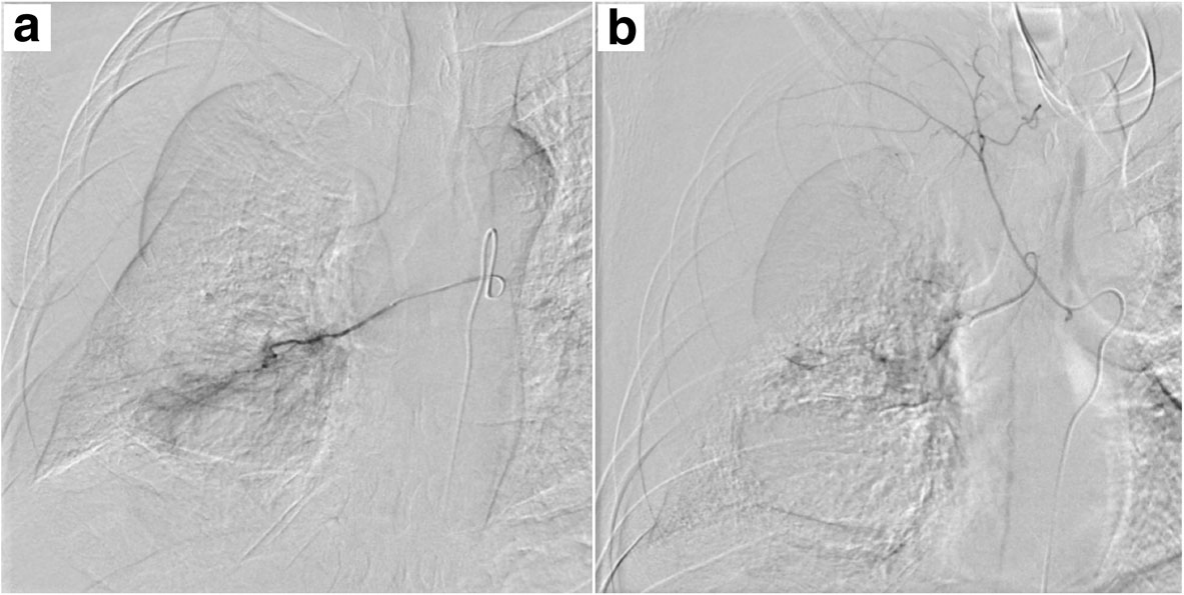
Findings of angiography of the bronchial artery. The descending aorta, and the superior and inferior branches of the right bronchial arteries were selectively imaged. The inferior branch of the bronchial artery (**a**) and another branch from the superior branch of the right bronchial artery (**b**) were thought to supply blood to the tumor and were embolized

The patient was a 44-year-old man, non-smoker, with a history of IgA nephropathy. He had no history of trauma or anticoagulant use and had no significant/relevant family history. He had no smoking history. The patient was referred to our hospital with the chief complaint of bloody sputum. He also complained of right upper back pain. He was alert, afebrile, and his vital signs were stable, with a peripheral arterial oxygen saturation level of 98% on room air. A plain chest X-ray revealed decreased radiolucency of the right lung field and an opacity in the right lower lung field (Fig. 1a). Chest computed tomography revealed a right pleural effusion and a large round mass in the right lower lobe, measuring 6.7 × 5.6 cm in size (Fig. 2a, b). This mass was not observed in a CT examination performed 10 months previously (Fig. 2c). Hematological examination showed a leukocyte count of 8200/μL, hemoglobin level of 12.3 g/dL, serum creatinine of 1.86 mg/dL, serum C-reactive protein level of 0.19 mg/dL, prothrombin time of 10.5 s, activated partial thromboplastin time of 25.9 s, serum fibrinogen level of 412 mg/dL, and a serum D-dimer level of 0.8 mg/L. Tests performed for markers of infectious diseases, such as serum β-D-glucan, serum aspergillus galactomannan antigen, and serum anti-glycopeptidolipid core immunoglobulin A antibodies were all negative. A test for tuberculosis-specific interferon-gamma and sputum smear examination for acid-fast bacilli were negative. We performed chest tube drainage of the right thorax and confirmed bloody pleural effusion. The chest tube drained 600 mL of bloody effusion within 2 h. Six hours after admission, as the patient continued to have hemoptysis and his hemoglobin level dropped from 12.3 to 10.3 g/dL, emergency bronchial artery embolization was performed (Fig. 3).

On the following day, the chest X-ray findings deteriorated (Fig. 1b) and the hemoglobin level dropped further from 10.3 to 7.4 g/dL. The hemoptysis persisted, and there was no reduction in the drainage volume of the bloody fluid from the chest tube. Therefore, we decided to perform emergency surgery.

**Fig. 4:**
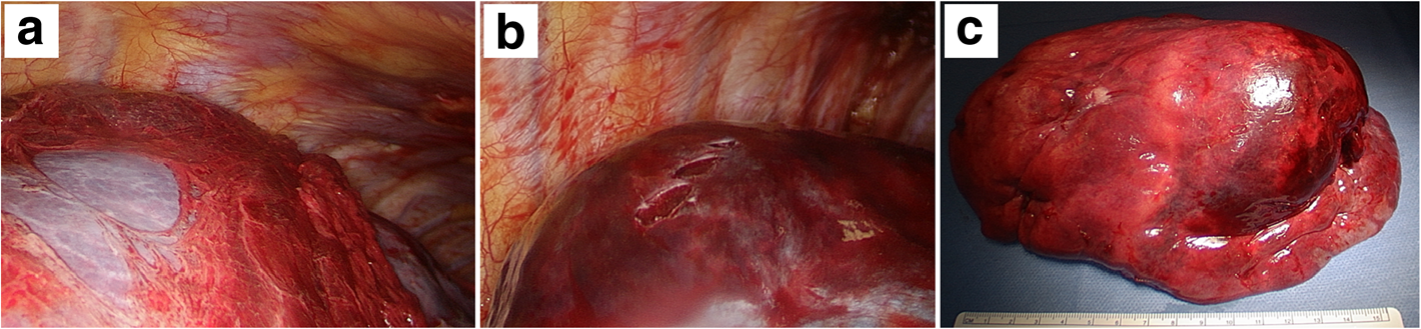
Intraoperative findings. Bloody pleural effusion, a fibrin clot on the lung surface, and a giant mass in the right lower lobe were observed. There was evidence of injury to the visceral pleura (**a**, **b**). Resected right lower lobe. The gross size of the tumor was 9.0 × 8.0 cm (**c**)

**Fig. 5:**
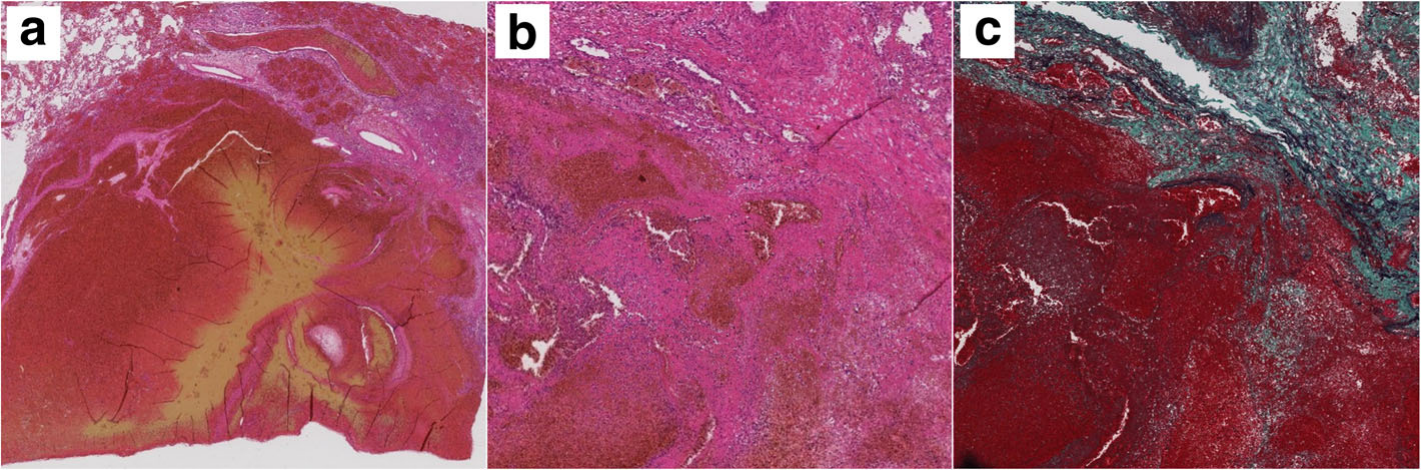
Histopathological examination showed a hematoma and rupture of a blood vessel wall within the hematoma. **a** HE-stained section under low magnification. **b** HE-stained section under high magnification. **c** Elastica van Gieson’s stain

**Fig. 6:**
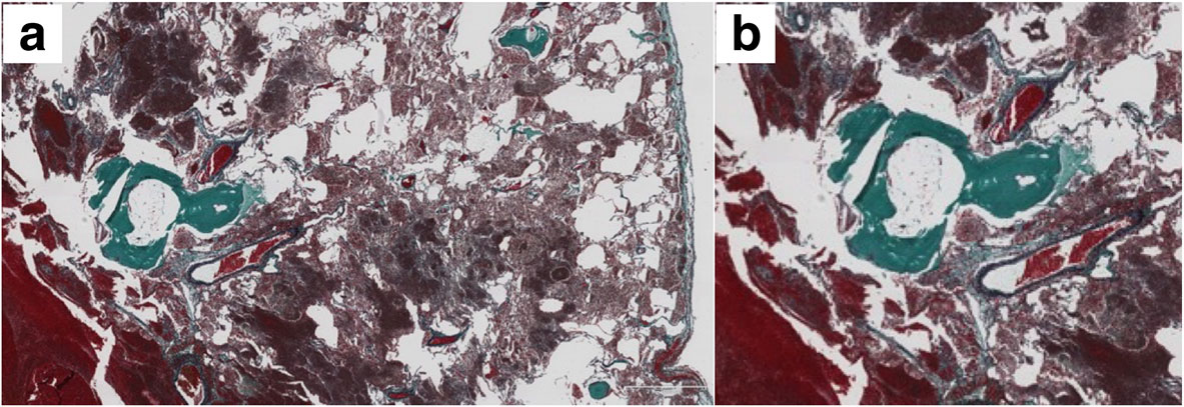
Multiple ossified areas with bone marrow formation are observed around the hematoma. **a** Elastica Masson-Goldner-stained section under low magnification. **b** Elastica Masson-Goldner-stained section under high magnification

Intraoperative observation showed bloody pleural effusion, fibrin clots on the lung surface, and a giant mass in the right lower lobe. There was evidence of injury to the visceral pleura, which was stretched excessively by the mass (Fig. 4). We performed right lower lobectomy with extirpation of the clot. We could not exclude the possibility of lung cancer; therefore, we additionally performed lymph node dissection. The operative time and estimated blood loss were 143 min and 163 mL, respectively. Histopathological examination showed a hematoma with granulation tissue and multiple ossified areas with bone marrow formation. The hematoma, which measured 9.2 × 8.0 cm in maximum diameters, consisted of fresh and old hemorrhages, and rupture of a blood vessel wall was observed within the hematoma (Fig. 5). In addition, multiple ossified areas with bone marrow formation were observed around the hematoma. There was no evidence of malignancy or angiitis. Based on these findings, we diagnosed this patient as having a pulmonary hematoma with diffuse dendriform pulmonary ossification (Fig. 6). After the operation, the patient was discharged without any complications, and a follow-up chest X-ray showed no evidence of recurrence.

## Discussion

Pulmonary hematoma is defined as the presence of blood within the alveolar and interstitial spaces [1]. In general, the pulmonary hematoma is caused by chest trauma. However, rare cases without a previous history of trauma have been reported, when the condition is referred to as spontaneous or idiopathic pulmonary hematoma. Anticoagulant therapy, thrombocytopenia, and rare diseases such as Ehlers-Danlos syndrome are some of the presumed causes of spontaneous or idiopathic pulmonary hematoma [1–7]. In our case, there was no history of trauma and no history of anticoagulant therapy, and his coagulation profile was normal. In addition, the pathogenesis remained unclear. Therefore, we diagnosed the case as a rare case of spontaneous pulmonary hematoma.

A noteworthy feature of this patient was that the histopathology revealed multiple ossified areas with bone marrow formation within the hematoma. Such a condition is called diffuse pulmonary ossification (DPO). While this condition has not previously been reported to be associated with intrathoracic bleeding, there is a possibility that it was associated with the intrathoracic bleeding in some way in our present case.

DPO is a rare disease that is characterized by diffuse and ectopic ossification in the lung tissue, and was first reported by Luschka in 1856 [8]. DPO has been classified into two subtypes: the nodular and dendriform types of DPO. Of the two types, dendriform pulmonary ossification is the less common. Nodular DPO usually occurs in situations of chronic congestion, such as mitral valve stenosis. On the other hand, dendriform DPO occurs secondary to acute or chronic damage of the lung, such as idiopathic pulmonary fibrosis, acute respiratory distress syndrome, and chronic obstructive pulmonary disease [9, 10]. In the case reported herein, the ossification was of the dendriform type; however, no abnormalities were observed in the background lung, and the cause of the ossification remained unclear. Since DPO is usually asymptomatic, as per most previous case reports, the condition is usually discovered accidentally at autopsy and rarely during the life of the patient. Furthermore, a literature search to the best of our ability revealed no previous report of pulmonary hematoma with DPO associated with intrathoracic bleeding requiring emergency surgery, as in this case; therefore, this was a very rare and interesting case. We think that the hemorrhage continued to occur as a result of a vessel rupture, so that the hematoma increased in size and the visceral pleura tore due to the pressure exerted by the hematoma.

Patients with pulmonary hematoma without a previous history of trauma are often asymptomatic; however, some cases may present with symptoms such as hemoptysis [11].

Because pulmonary hematoma is visualized as an opacity on imaging examinations, it is often difficult to distinguish it from malignancy. However, pulmonary hematoma usually disappears spontaneously from 3 weeks to 3 months, in the absence of any special complications [7, 11]. Therefore, in the absence of any serious symptoms and the presence of sufficient evidence to suspect pulmonary hematoma, such as a previous history of trauma, the management would be conservative. It is important to conduct further evaluation for the possibility of malignancy if the opacity is found to increase in size on follow-up examinations. In addition, in recent years, chest magnetic resonance imaging has also been shown to be useful for the diagnosis of pulmonary hematoma, and especially for distinguishing hematoma from malignancy [12].

No specific treatment or management for DPO has been established, but in asymptomatic cases, or aggressive treatment is necessary. Regular follow-up with respiratory function testing and imaging examinations is recommended [9].

The patient reported here is currently under observation as an outpatient, and a follow-up chest X-ray has shown no evidence of recurrence. However, as the cause remains unclear, bearing in mind the possibility of appearance of new lesions/recurrence, we propose to keep the patient under careful follow-up.

## Conclusion

We have reported a very rare and interesting case of spontaneous pulmonary hematoma associated with diffuse pulmonary ossification that required emergency surgery.

## Abbreviation

DPO: Diffuse pulmonary ossification
